# Motion matters: the role of milling ball trajectories in mechanochemical reactions

**DOI:** 10.1039/d5mr00112a

**Published:** 2025-12-02

**Authors:** Marisol Fabienne Rappen, Justus Mäder, Sven Grätz, Lars Borchardt

**Affiliations:** a Ruhr-Universität Bochum Universitätsstraße 150 Bochum 44801 Germany Lars.Borchardt@rub.de

## Abstract

Mechanochemistry has become a powerful and sustainable approach in synthetic chemistry, yet the fundamental principles governing energy transfer during milling remain poorly understood. In particular, the trajectory of the milling ball has been largely overlooked in mechanistic studies. To address this, we employed high-speed recordings to precisely track ball motion, enabling accurate calculation of kinetic energies and their comparison with theoretical values. The use of hollow and solid balls of varying sizes further allowed us to disentangle the effects of altered trajectories in both the Finkelstein reaction and the direct mechanocatalyzed Suzuki coupling. This work underscores the critical importance of milling ball trajectory in mechanochemistry and highlights the need to consider this parameter in future mechanistic studies and in the development of optimized milling protocols.

## Abstract 2

In this work, high-speed imaging reveals how milling ball trajectories control energy transfer and reaction efficiency in mechanochemistry, providing a basis for improved reaction design and reproducibility.

## Graphical abstract

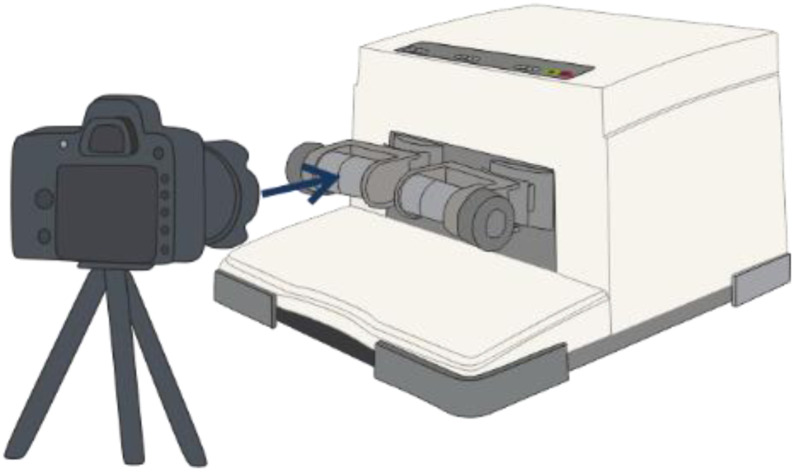

## Introduction

Mechanochemistry has gained significant attention in recent years as a sustainable and efficient method for driving chemical transformations. Its applications span diverse fields including materials science, organic synthesis, and pharmaceutical manufacturing, where it has emerged as a viable alternative to conventional solution-based methods.^1–5^ By operating under solvent-free or solvent-minimized conditions, mechanochemical approaches offer notable environmental advantages while enabling precise control over reaction conditions.^6–8^ Parameters such as milling frequency, duration, and vessel design have been extensively studied and optimized to enhance reaction outcomes.

Despite this progress, the influence of one critical parameter remains comparatively underexplored: the movement trajectory of the milling balls. The trajectory directly impacts the nature and magnitude of mechanical forces applied to the reactants, including impact frequency, contact time, and energy transfer efficiency. Understanding these dynamics is particularly crucial in mechanochemical processes where localized energy inputs govern the course and selectivity of the reaction.

To address this gap, the present study focuses on single-ball milling systems, which represent the dominant setup in organic mechanochemistry conducted with mixer mills—excluding special cases such as piezoelectric systems that utilize large numbers of microbeads.^9–17^ These single-ball systems not only allow for clearer mechanistic interpretation but are also widely employed in practical synthesis. Furthermore, differences in milling trajectories depending on the design of commercial milling devices (*e.g.*, Retsch *vs.* SPEX *vs.* Fritsch) suggest that trajectory-dependent effects may have broader implications across platforms. Particularly in direct mechanocatalytic processes, where catalysis occurs at the surface of the milling ball^18–21^ the physical interaction between the ball and the vessel plays a decisive role. Thus, experimentally resolving and quantifying the trajectory becomes essential for rationalizing observed reactivity and guiding the design of improved protocols. Recent developments in *operando* analysis, including acoustic monitoring and high-speed imaging, have demonstrated the feasibility of correlating ball motion with reaction progress and polymorphic transformations in a Fritsch Pulverisette-23 mixer-mill.^22^

**Fig. 1:**
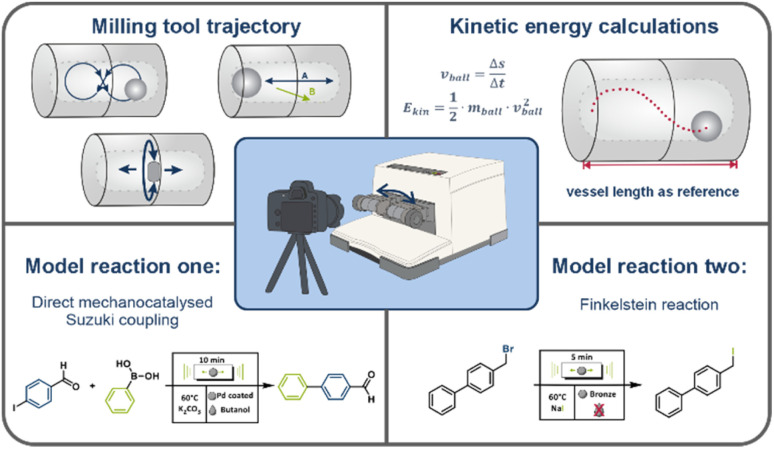
Scope of this study: high-speed camera recordings of the milling tools used inside a PFA vessel milled in a MM500 mixer mill by Retsch at different milling frequencies. The recordings enabled calculation of the kinetic energies through tracking of the ball's trajectory for 500–1000 separate frames through tracker video analysis, and the two model reactions conducted, namely the Finkelstein reaction and the direct mechanocatalysed Suzuki coupling.

In this work, we employ high-speed video analysis to systematically study the motion patterns and kinetic energies of milling tools under realistic reaction conditions (Fig. 1). Compared to purely theoretical estimates,^23–26^ this approach provides more accurate and experimentally grounded insights. Two representative model reactions—a Finkelstein reaction and a direct mechanocatalysed Suzuki coupling—are used to demonstrate the influence of milling tool trajectory on chemical reactivity. Our results underscore that the ball trajectory is not merely a secondary physical parameter but a key mechanistic variable in mechanochemistry. It should be considered alongside more established factors such as milling frequency and duration in both the interpretation and optimization of mechanochemical processes.

## Results and discussion

**Fig. 2:**
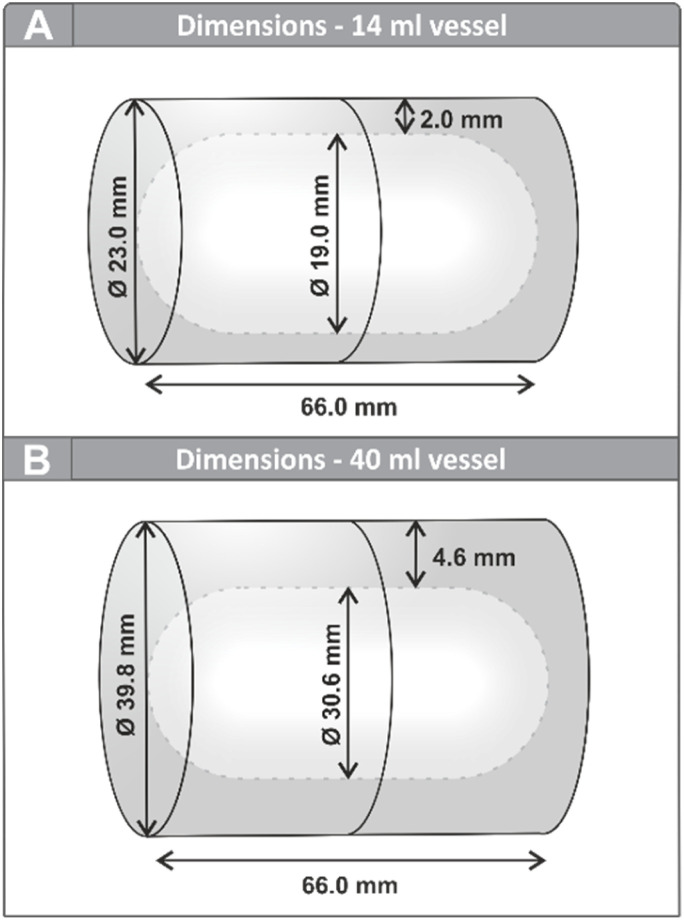
Vessel dimensions of a 14 mL vessel (A) and a 40 mL vessel (B) manufactured in the in-house tool shop.

During previous experiments on direct mechanocatalysis,^19^ we repeatedly observed that the reaction powder was not primarily concentrated at the ends of the milling vessel—as might be expected if the milling ball were moving back and forth between the vessel walls—but rather finely smeared along the side walls. This unexpected distribution raised questions about the actual movement pattern of the milling ball within the vessel. Preliminary slow-motion recordings using a smartphone camera suggested that, contrary to common assumptions, the ball did not travel in a linear path between the vessel ends but instead followed a more complex trajectory; instead of discrete impacts, a more continuous rolling motion was observed. To investigate this observation in more detail and under controlled conditions, we conducted high-speed video recordings using a Sony RX100 VII camera operating at 1000 frames per second. All recordings were conducted using a MM500 mixer mill by Retsch. For these studies, thin-walled semi-transparent 14 mL PFA (perfluoroalkoxy polymer) vessels were used to allow visual access to the ball's motion without significantly altering the mechanical properties of the system. The dimension of our standard vessel design is depicted in Fig. 2A. Bronze balls with diameters of 10 mm, 15 mm, and 20 mm were employed in this study to observe changes in movement patterns for the differently sized balls. Bronze was selected as its superior weldability, compared to our commonly used 1.3505 steel, was crucial for manufacturing hollow balls of the mentioned sizes, while still providing sufficient durability of the metal coating. To maintain comparability, the solid balls were likewise produced from bronze. To investigate the influence of milling tool geometry on the motion path, two differently shaped cylinders were fabricated in the in-house tool shop. Both cylinder shapes were produced in two different sizes and made from 1.3505 steel. We further investigated the influence of a larger vessel on the ball's movement, whereby in case of this vessel with a volume of 40 mL (Fig. 2B), a transparent PMMA (polymethyl methacrylate) version was used instead, as the thicker PFA walls impeded observation. While this setup did not allow precise energy calculations for the larger vessel due to differences in material properties, it provided a sufficient basis for qualitative comparison of ball trajectories and relative kinetic energies.

### Ball movement in an empty vessel

**Fig. 3:**
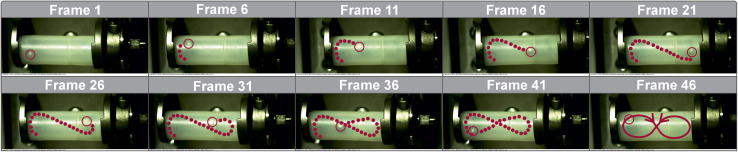
Series of frames to depict the movement of a 10 mm ball inside a 14 mL PFA vessel. Every fifth frame was used; the position of the ball in the previous frames is indicated by red dots. The ball is outlined in red in the current frame. In the last frame (frame 46), the movement is shown again schematically.

**Fig. 4:**
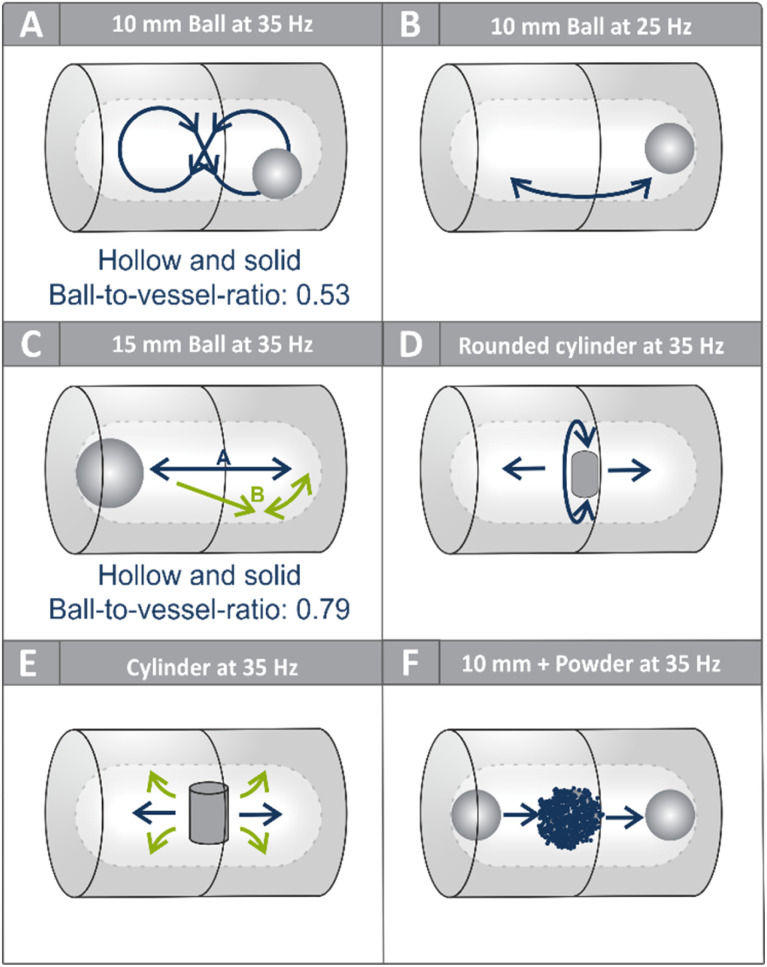
(A) Main movement observed of a 10 mm ball at 35 Hz. The ball mostly follows a figure-eight shaped path. (B) The main movement of a 10 mm ball at lower frequencies *e.g.*, 25 Hz. The ball is mostly scratching the vessel walls in the lower part of the vessel, as its velocity is not high enough to follow the figure-eight shaped path continuously (C) two main observed movements of a 15 mm ball at 35 Hz, which are a movement in a straight line from one vessel end to the other, whereby the ball is oftentimes in contact with the forward-facing vessel wall and a rolling motion occurring in the vessel ends occasionally. (D) Rounded cylinder at 35 Hz. Besides scratching the forward-facing vessel wall, the cylinder oftentimes tilts 90° in the *z*-axis, so the rounded ends are in contact with the forward and backward-facing vessel wall. This was observed for both sizes of cylinders used. (E) Main movements of a cylinder inside a milling vessel. It is occasionally observed that the cylinder is scratching on the forward-facing vessel wall, but it is oftentimes distracted from this path. The contact with vessel walls is lower compared to the balls and the other type of cylinder. (F) Main movement of a 10 mm ball with added reaction mixture immediately after starting the mill.

As noted above, the 10 mm milling ball at a frequency of 35 Hz consistently exhibited a pronounced figure-eight motion, predominantly along the forward-facing wall of the vessel (Fig. 3 and 4A). The term “forward-facing wall” refers to the front wall of the milling jar facing the camera (SI, Chapter 5.1 and Fig. S3). This trajectory is a consequence of the angled oscillatory motion of the mixer mill and suggests that frictional forces dominate under these conditions. Occasional deviations from this path—such as sporadic impacts with the cylindrical vessel walls—were observed, but these were relatively rare at higher frequencies. Given the strong influence of the trajectory on energy transfer, we systematically investigated how changes in milling frequency affect ball motion. As the frequency decreased, the figure-eight pattern became increasingly unstable. At lower frequencies, the ball exhibited more linear trajectories, intermittent levitation near the center of the vessel, and frequent back-and-forth rolling near the vessel base (Fig. 4B). These changes reflect a shift from a friction-dominated regime to one governed more by impact dynamics, particularly with the vessel ends.

A second critical factor influencing the trajectory is the ratio between the ball diameter and the vessel diameter. When the ball diameter was increased to 15 mm—while the vessel dimensions remained unchanged—the figure-eight motion was no longer observed (Fig. 4C). The reduced free volume limited the ball's movement, leading to a more linear path with only minor rolling along the curvature of the vessel ends or the forward-facing wall. This trajectory shift is not simply a geometric curiosity; it highlights the mechanical limitations imposed by increasing ball size relative to vessel dimensions. A 14 mm milling ball, only about 1 mm smaller than the previously used 15 mm ball, already exhibits a pronounced figure-eight trajectory in the same milling vessel. The critical point at which the motion appears to change therefore lies between a ball-to-vessel ratio of approximately 0.73 and 0.79. This highlights the importance of explicitly reporting the ball-to-vessel size ratio in mechanochemical studies, as even such minor size differences can strongly influence the mode of energy transfer and thus potentially the outcome of chemical transformations. Despite its potential impact on reproducibility and scalability, this parameter has received little attention in the literature and is often omitted entirely. Future work in the field should take care to report this ratio and consider its effects during reaction optimization and scale-up.

The hypothesis that the ball-to-vessel diameter ratio governs the available movement patterns is further supported by observations made in a larger vessel with a volume of 40 mL. In this setup, both 15 mm (ball-to-vessel ratio: 0.49) and even 20 mm (ball-to-vessel ratio: 0.65) milling balls regained the ability to follow a figure-eight trajectory—similar to that observed for 10 mm balls in smaller vessels. This reinforces the conclusion that spatial freedom, rather than absolute ball size, is the key determinant of the trajectory.

To isolate the role of mass from that of geometry, we conducted additional experiments using hollow balls with diameters of 10 mm, 15 mm, and 20 mm. Across all sizes tested, the hollow milling balls displayed motion patterns nearly identical to their solid counterparts. These results suggest that mass primarily influences the kinetic energy of the ball, while the geometry and ball-to-vessel ratio remain the dominant factors controlling the trajectory.

To further explore how milling tool shape affects the movement, we replaced spherical balls with cylindrical tools—both regular cylinders and cylinders with rounded ends—across two different sizes. In both cases, the rounded cylinders exhibited frequent sliding along the forward-facing wall of the vessel, with their ends maintaining regular contact with the surface (Fig. 3D). These trajectories were indicative of friction-dominated motion. However, the contact frequency was notably lower than that observed for the 10 mm milling balls, implying reduced mechanical energy transfer per unit time. Regular (flat-ended) cylinders showed even less vessel contact, and in the case of the larger flat-ended cylinder, jamming events were frequently observed, rendering the tool effectively immobile (Fig. 3E).

Building on these insights, we also investigated the influence of the vessel geometry itself. A modified vessel design with flat inner ends was fabricated to examine its effect on the motion pattern (SI, Chapter 1.1 and Fig. S1). Under otherwise identical conditions (10 mm ball, 35 Hz), the figure-eight trajectory was still observed, but with a noticeably lower contact frequency compared to the standard vessel design. This finding suggests that even seemingly minor geometric changes can impact the movement behavior and, consequently, reaction efficiency. A similar approach was recently reported by Bolm *et al.*, in which 18 different vessel shapes were 3D printed and the motion was tracked using high-speed photography.^27^

Nonetheless, using a commercially available 14 mL PMMA vessel by Retsch showed that small variations in internal curvature—such as those introduced by routine design differences—did not produce measurable effects on ball trajectory. Taken together, these results highlight the importance of not only the milling ball and its ratio to the vessel, but also the broader design choices regarding tool shape and reaction vessel architecture. Even subtle geometric constraints can alter the dynamics of energy input and thus influence reaction outcomes—factors that must be carefully considered when comparing or reproducing mechanochemical experiments across different setups.

### Ball movement in the presence of reactants

All previously described recordings were performed in the absence of any reaction mixture, allowing for a direct observation of ball motion uninfluenced by substrate interactions. To evaluate the effect of a typical reaction medium on milling ball trajectories, additional high-speed recordings were carried out at 35 Hz using a standard reaction mixture comprising solid substrates, a bulking agent, and a small amount of liquid additive (LAG). Immediately upon initiating milling, the motion of the 10 mm ball shifted notably: instead of following the previously observed figure-eight trajectory, the ball moved back and forth between the ends of the vessel, piercing through the powder bed, which was initially concentrated in the center (Fig. 4F). At this early stage, the solid reactants were not yet homogeneously mixed, and a large portion remained suspended loosely in the middle of the vessel. This disrupted the smooth rolling trajectory observed in the empty system. It is also conceivable that the mill had not yet fully reached its steady-state operating frequency, potentially contributing to the deviation. As milling progressed, mixing improved and the powder began adhering to both the vessel walls and the milling ball. Within approximately 20–30 seconds, the mixture transformed into a uniform slurry, enabling a return to the characteristic figure-eight movement. These observations underscore the critical role of rheological evolution during milling and may help explain why liquid-assisted grinding (LAG) often improves reactivity: it not only enhances substrate wetting but also supports ball motion conducive to efficient energy transfer.

To probe this effect further, a similar experiment was performed using only dry solid reactants, without the addition of LAG. The initially loose powder once again disrupted the ball's movement, but with continued milling, the powder compacted onto the vessel walls. This facilitated the re-emergence of the figure-eight trajectory, albeit after a longer induction time. These findings suggest that both the transient physical state of the reaction mixture and its rheology over time significantly influence ball dynamics. Furthermore, this may indicate an influence of the milling ball trajectory on the mixing efficiency. To directly investigate the effect of filling degree, further recordings were conducted using potassium carbonate (K_2_CO_3_) as a model bulking agent. One 10 mm milling ball was used in each case, and the vessel was filled with 1 g (2.95%), 2 g (5.88%), 3 g (8.82%), and 5 g (14%) of K_2_CO_3_ by volume. These recordings were taken after ∼30 seconds of milling, by which time the mixture was assumed to have reached a relatively stable distribution. At low filling degrees (1–2 g), which are common in mechanochemical protocols, the figure-eight trajectory was still observable, although occasional interruptions in the motion were noted. At higher filling levels (3–5 g), the ball's motion became increasingly difficult to track—particularly along the forward-facing vessel wall, where the figure-eight pattern is typically most visible due to the angular motion of the vessel holder. This strongly indicates that, beyond a certain threshold, the dense powder bed restricts the spatial freedom of the ball, suppressing its ability to follow the characteristic trajectory.

Taken together, these results highlight the central role of filling degree and reaction mixture rheology in governing milling ball motion. Both parameters can significantly alter energy transfer dynamics and thus must be carefully considered when designing or reproducing mechanochemical reactions. Moreover, their influence on the trajectory may partially account for differences in reaction efficiency across studies employing similar setups but varying material loads.

### Kinetic energy calculations

In recent years, several approaches have been proposed to estimate the kinetic or transferred energy in ball mills.^28–30^ Recent modeling work has further emphasized the importance of understanding ball motion and energy dissipation, for example by describing tangential and normal collision dynamics through discrete element method (DEM) simulations.^28^ These studies highlight that the trajectory and contact behaviour of the milling balls are key determinants of energy transfer efficiency. Nevertheless, most available models rely on simplifying assumptions and do not fully reproduce experimental reaction conditions. Quantifying the energy input therefore remains a central challenge in mechanochemistry, as it is key to understanding reactivity, reproducibility, and scalability. Our high-speed video recordings enabled direct experimental tracking of the milling ball's velocity, and hence its kinetic energy, under realistic conditions. Using tracker video analysis, ball positions were tracked across 500–1500 consecutive frames at various milling frequencies. The vessel length was used as the calibration reference. Mean velocities were calculated from the displacement between successive frames and the known frame rate, and these were subsequently used to calculate kinetic energies. The results were compared with theoretical values derived from two idealized motion patterns—a straight-line and a figure-eight trajectory. Further details and associated limitations are discussed in the SI (Section 5.2).

**Fig. 5:**
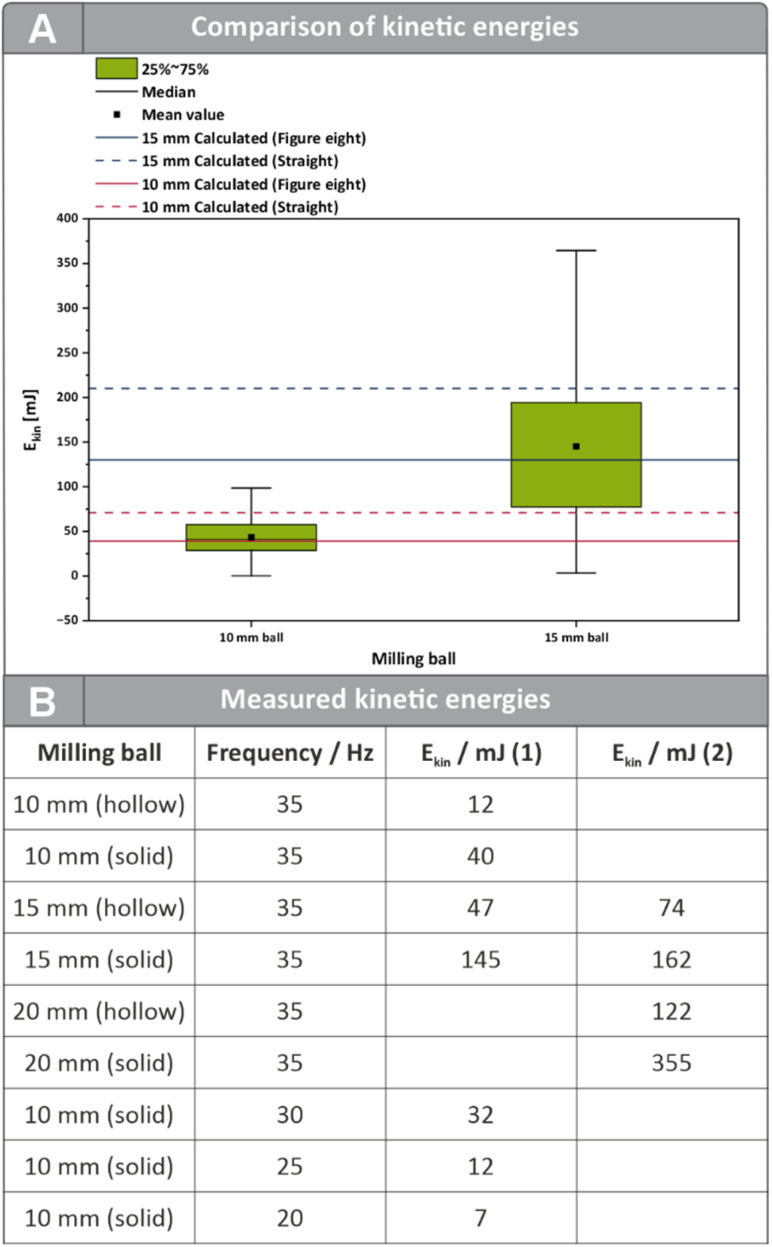
(A) Comparison of the kinetic energies derived from the high-speed recordings. The distances between two ball positions were measured and the time was calculated through the number of frames and the known framerate used for recording. Furthermore, the idealized values were determined theoretically by calculations. These are depicted in the SI in more detail (SI, Chapter 5.2). (B) Kinetic energies obtained from the high-speed recordings. (1) Kinetic energies measured inside a 14 mL vessel. (2) Kinetic energies inside a 40 mL vessel.

For the 10 mm ball, experimentally determined kinetic energies showed substantial variation (Fig. 5A), which can be attributed to the inclusion of all observed motions—including deviations from the ideal figure-eight path. The experimentally obtained average energies for the 10 mm ball are in close agreement with the theoretical estimates for the figure-eight trajectory, despite the latter neglecting frictional effects. This suggests that the simplified model provides a reliable approximation of the dominant energy transfer processes for the 10 mm ball in the mixer mill (Fig. 5A). The comparison with the straight-line model is particularly revealing. This motion becomes more prevalent at lower milling frequencies or when larger balls are used. The kinetic energy calculated for a 10 mm ball moving linearly at 35 Hz was significantly higher than that observed experimentally. This reinforces the notion that theoretical models must account for actual trajectory patterns to accurately reflect energy transfer.

For the 15 mm ball, the experimentally measured kinetic energies were substantially lower than those predicted for an idealized straight-line motion as well. In reality, the ball does not move in a perfectly straight path but exhibits rolling and directional changes within the vessel. In contrast, the theoretical figure-eight trajectory values are in close agreement with the experimental data. This suggests that the figure-eight model better reflects the influence of rolling and directional changes on energy transfer, even without explicitly accounting for dissipative effects. These findings highlight that simplified models can underestimate the effects of rolling and directional changes on energy transfer, underlining the importance of considering the ball trajectory in mechanochemical modelling.

Finally, we observed that the mean kinetic energy obtained by the high-speed recordings did not increase linearly with milling frequency (SI, Chapter 5.1 and Figure S5). This non-linear relationship suggests that higher frequencies may not necessarily result in proportionally higher energy input, likely due to changes in the movement pattern—such as reduced contact time or frequency with the vessel walls. Overall, these findings strongly advocate for a trajectory-informed approach to energy estimation in mechanochemical systems, especially when aiming to interpret or compare experimental results across different setups.

### Trajectory and energy efficiency in model reactions

**Fig. 6:**
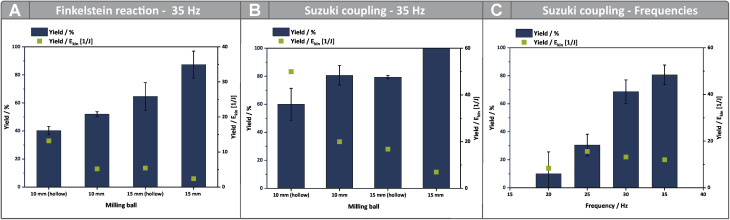
(A) Yields of the Finkelstein reaction conducted in a MM500 mixer mill by Retsch as well as the yields normalized per Joule of the kinetic energy calculated using the high-speed recordings. The reactions were conducted at 35 Hz and 60 °C to ensure near-isothermal conditions. A 14 mL PFA vessel containing the milling tool and the reaction mixture (247 mg 4-Bromomethyl-1′,1′-Biphenyl and 449 mg Sodium Iodide) was utilized. (B) Yields of the Suzuki coupling conducted in a MM500 mixer mill by Retsch*,* as well as the yields normalized per Joule of kinetic energy obtained by the high-speed recordings. The reactions were conducted at 35 Hz and 60 °C to ensure near-isothermal conditions. A 14 mL PA 6 (polyamide 6) vessel containing the milling material and the reaction mixture (1 g K_2_CO_3_, 122 mg phenylboronic acid, 232 mg iodobenzene, 311 µl butanol) was utilized. (C) Comparison of the yields, as well as the yields normalized for the kinetic energies at different milling frequencies of the Suzuki reaction. The reactions were conducted at 60 °C to ensure isothermal conditions. A 14 mL PA vessel containing the milling material and the reaction mixture (1 g K_2_CO_3_, 122 mg phenylboronic acid, 232 mg iodobenzene, 311 µl butanol) was utilized.

To assess how milling ball trajectory influences reactivity, we applied our kinetic and motion analyses to two model reactions: the Finkelstein reaction of 4-bromomethyl-1′,1′-biphenyl with sodium iodide, and the direct mechanocatalytic Suzuki coupling of 4-iodobenzaldehyde with phenylboronic acid.^19,31^ These reactions were selected to represent distinct mechanistic classes—solution-like diffusion *vs.* surface-bound catalysis—and to test whether the impact of trajectory holds across different contexts. Reactions were performed using hollow and solid milling balls (*Ø* 10 mm and 15 mm), and the yields were normalized to the mean kinetic energy determined from the high-speed video recordings (Fig. 6). This allowed a comparison of reaction efficiency per unit of energy input, decoupled from the effects of ball size or mass.

In both reactions, the hollow 10 mm ball showed surprisingly high efficiency relative to its lower kinetic energy. This is attributed to longer contact times and more continuous interaction with the vessel surface—consistent with a friction-dominated figure-eight trajectory. In contrast, the solid 15 mm ball, despite having significantly higher kinetic energy, exhibited a more constrained movement and shorter contact times, leading to a lower efficiency per joule. These findings highlight that contact dynamics—not just energy magnitude—drive reactivity. The Suzuki coupling, which occurs at the milling tool surface, amplified these differences. The hollow 10 mm ball outperformed larger or heavier counterparts in terms of energy-normalized yield, underscoring the importance of high-frequency surface contact in direct mechanocatalysis.

In a larger 40 mL vessel (SI, Chapter 7 and Fig. S7), 15 mm and 20 mm balls regained the ability to perform figure-eight trajectories. However, the solid 20 mm ball was less efficient than the 15 mm or hollow 20 mm balls—despite its higher kinetic energy—likely due to excess velocity reducing contact time. This again confirms that trajectory and its impact on contact quality are central to reactivity.

Finally, variation of milling frequency revealed a non-linear relationship between energy input and reaction efficiency (Fig. 6C). For example, the 10 mm ball was equally efficient at 30 Hz as at 35 Hz, likely due to similar movement, contact times and durations. At 20 Hz, efficiency declined as the figure-eight trajectory became less pronounced, further highlighting the role of ball trajectory in governing energy transfer dynamics.

Taken together, these results validate the earlier mechanistic insights and demonstrate that ball trajectory governs not just the magnitude, but the quality of energy transfer in mechanochemical reactions. This finding is particularly crucial in direct mechanocatalysis, where surface interaction is central to reactivity.

## Conclusions

In this study, we demonstrated that the trajectory of the milling ball—an often-overlooked parameter in mechanochemistry—plays a central role in determining the efficiency and nature of energy transfer during ball milling reactions. Using high-speed video recordings, we systematically analyzed the motion of different milling tools under realistic conditions, revealing that a standard 10 mm ball at 35 Hz predominantly follows a figure-eight trajectory, driven largely by frictional interactions with the vessel wall.

This movement pattern was associated with increased contact frequency and duration, which significantly enhanced reaction efficiency—even when the kinetic energy was lower than that of larger or heavier balls. In contrast, increasing the ball diameter or decreasing the milling frequency disrupted the figure-eight trajectory, leading to more impact-driven motion with reduced contact times. These altered dynamics correlated with reduced energy efficiency in model reactions, reinforcing the importance of trajectory as a mechanistic variable.

We further quantified kinetic energies from experimental ball velocities and compared them with theoretical energy estimates based on idealized movement patterns. The comparison revealed that simplified models—particularly those ignoring real trajectories—fail to capture the true dynamics of energy input, often under- or overestimating energy transfer depending on the assumed motion. This mismatch underscores the need for trajectory-aware energy models in mechanochemistry.

To assess the chemical relevance of these findings, we applied them to two representative reactions: a Finkelstein reaction and a direct mechanocatalytic Suzuki coupling. In both cases, the results supported the hypothesis that efficient energy transfer depends not only on the magnitude of kinetic energy, but critically on the nature of the ball's interaction with the reaction medium and vessel surface. Notably, when yields were normalized to the kinetic energy input, smaller balls following figure-eight trajectories outperformed larger or heavier tools—highlighting the role of contact dynamics over brute force.

Additional variables, such as filling degree, reaction mixture rheology, vessel geometry, and milling frequency, were also shown to influence the observed trajectory and, by extension, reaction outcomes. These results emphasize that subtle changes in system design can lead to significant differences in mechanochemical behavior—raising important questions for reproducibility, scalability, and method standardization across different laboratories and setups.

In summary, our findings establish that the milling ball's trajectory is a critical parameter in mechanochemical reactivity, influencing not only how energy is delivered, but how effectively it is transferred to the reacting system. As such, trajectory should be considered alongside conventional factors such as ball size, mass, and surface area. Incorporating this parameter into mechanistic models, experimental reporting, and instrument design will improve the reliability and optimization of mechanochemical protocols across the field.

## Author contributions

Marisol Fabienne Rappen: conceptualization, data curation, formal analysis, investigation, methodology, verification, visualization, writing – original draft. Justus Mäder: formal analysis, investigation, verification. Sven Grätz: conceptualization, data curation, funding acquisition, project administration, resources, supervision, writing – review & editing. Lars Borchardt: conceptualization, data curation, funding acquisition, project administration, resources, supervision, writing – review & editing.

## Conflicts of interest

There are no conflicts to declare.

## Supplementary Material

MR-003-D5MR00112A-s001

MR-003-D5MR00112A-s002

MR-003-D5MR00112A-s003

## Data Availability

The data supporting this article have been included as part of the supplementary information (SI). Supplementary information: detailed account of the calculated kinetic energies presented in this article, along with high-speed video recordings documenting the movement of the milling tools under distinct milling conditions. See DOI: https://doi.org/10.1039/d5mr00112a.
