# Supplementary material for: Motion matters: the role of milling ball trajectories in mechanochemical reactions
Source: RSC Mechanochem. 2025 Dec 2;3(2):235–42. doi: 10.1039/d5mr00112a (PMC12684371; doi:10.1039/d5mr00112a)
Supplement: MR-003-D5MR00112A-s001 [file MR-003-D5MR00112A-s001.pdf]

Supplementary Information (SI) for RSC Mechanochemistry.
This journal is © The Royal Society of Chemistry 2025

Supporting Information

Motion Matters: The Role of Milling Ball Trajectories in Mechanochemical Reactions

Marisol F. Rappen,[a] Justus Mäder [a], Sven Grätz [a], Lars Borchardt [a]

Table of contents

1. Eperimental section 2

 1.1 Used equipment and characterisation techniques 2

2. Synthetic procedures 3

 2.1 Direct mechanocatalysed Suzuki coupling 3

 2.2 Finkelstein reaction 3

3. Product characterization 4

4. High-speed recordings 5

 4.1 Equipment and Set-up 5

 4.2 Observed movements 5

5. Kinetic energy measurements and calculations 5

 5.1 Kinetic energies derived from high-speed recordings 5

 5.2 Calculated kinetic energies 7

  5.2.1 Idealized Figure of eight trajectory 7

  5.2.2 Idealized straight movement 8

6. Suzuki coupling PA vessel vs. PFA vessel 9

7. Suzuki coupling – 40 mL vessel 10

8. List of high-speed recordings 11

References 12

1. Eperimental section

1.1 Used equipment and characterisation techniques

General. All reagents were obtained from commercial suppliers at least in synthesis purity and used without further purification. The organic solvents utilised as LAG agents were purchased in analysis grade. For all experiments conducted, a MM500 Vario mixer mill (*Retsch*) was used. The milling vessels were made from PFA purchased from *Ammerflon*® GmbH and PA6 from *Technoplast.* The vessels were custom-made from the in-house tool shop through lathe processing. The solid and hollow bronze milling balls, as well as the different steel cylinders (1.3505 steel) were custom-made in the in-house tool shop as well. The milling tools´ masses, surface areas and kinetic energies derived from the high-speed recordings are given in Table S1. More information about the procedure of calculating the experimentally derived kinetic energies are given in Chapter 5.1.

Table S1: Properties of the differently sized hollow and solid milling balls, as well as cylinders used in the course of this work. Shown are the mass in g, the total surface area in mm^2 and the kinetic energies in mJ.

**Milling tool**	**Mass / g**	**A / mm^2**	**E_{kin} / mJ[a]**	**E_{kin} / mJ[b]**
		**10 mm ball**		
hollow	2.6	314	12	-
solid	4.6	314	40	-
		**15 mm ball**		
hollow	7.3	707	47	74
solid	15.8	707	145	162
		**20 mm ball**		
hollow	15.1	1257	-	122
solid	37.3	1257	-	355
		**Cylinder**		
small flat end	4.6	397	35[c]	-
small rounded	4.6	363	17[c]	-
large flat end	15.8	905	38[c]	-
large rounded	15.8	814	40[c]	-

[a] kinetic energy in the 14 mL vessel
[b] kinetic energy in the 40 mL vessel
[c] The kinetic energies may deviate from the presented results, since it is not always possible to strike the center of the cylinders due to their asymmetry and irregular motion.

The heating setup was custom made for the used *Retsch* MM500 mixer mill (left) using heating jackets from Ihne & Tesch (230-250 V, 300W, CrNi-Ni), as well as a temperature control panel (right) for setting the desired temperature. Previous measurements of the milling-induced temperature increase showed that the reaction mixture did not exceed 40°C under the applied conditions. To ensure that no sample experienced significantly higher temperatures and to maintain comparable conditions across all experiments, the external heating was set to 60 °C.

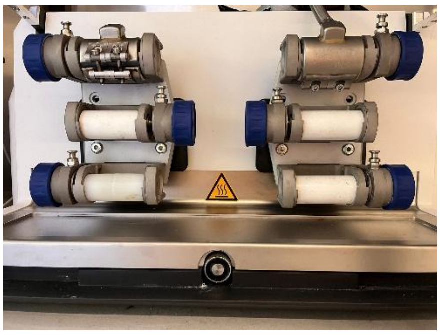

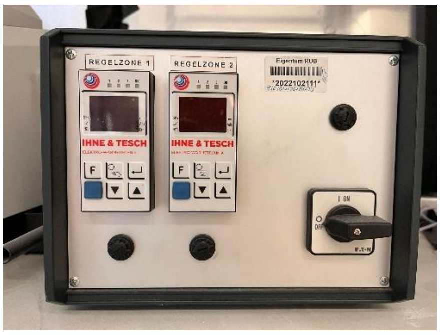

Modified vessel design. In this study, a modified vessel featuring flat inner ends instead of the standard curved geometry was designed for comparison with the conventional vessel. The design is illustrated in Figure S1.

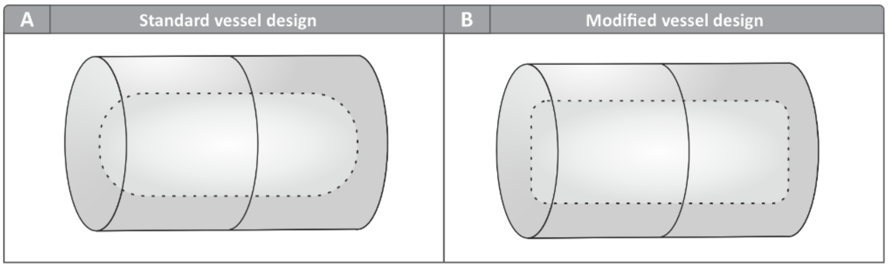

Figure S1: A) Our standard vessel design used in this study. This vessel features rounded ends, comparable to commercially available models. B) Modified vessel design with flattened inner ends. This vessel was designed to observe if larger design differences does change the movement pattern of the milling balls.

High performance liquid chromatography (HPLC) was performed on a shimadzu Nexera LC-40 lite. A Nucleodur C18, 3 µl Reversed phase column from Macherey-Nagel was used as stationary phase. An isocratic solvent mixture of 65 % acetonitrile, as well as 35 % water was used at a flow rate of 0.1 mL/min as mobile phase. The crude product (approx. 2 - 3 mg) was dissolved in 3 ml of the acetonitrile/water mixture (65:35) acidified with 5 % acetic acid, filtered through a syringe filter, and subsequently transferred to a HPLC vial.

Nuclear magnetic resonance spectroscopy (NMR). ^{1}H-NMR spectra were recorded in $CDCl_3$ on a Bruker Avance III HD spectrometer at 300 MHz. Spectra were referenced internally to residual solvent resonances and are reported relative to tetramethylsilane. Data are reported as follows: s = singlet, d = doublet, dd= doublet of doublets, m = multiplet; coupling constants in Hertz.

2. Synthetic procedures

2.1 Direct mechanocatalysed Suzuki coupling

For the standard reaction, phenylboronic acid (122 mg, 1 mmol, 1 eq.), 1 g of potassium carbonate (1 g, 7.3 mmol, 7.3 eq.), 4-iodobenzaldehyde (232 mg, 1 mmol, 1 eq.), as well as butanol (311 µl, η = 0.15, 3.4 mmol, 3.4 eq.) were placed in a 14 ml vessel made of perfluoroalkoxy polymer (PFA) or polyamide 6 (PA6) . Before the reaction mixture was added, a palladium-coated ball (Ø 10 mm or 15 mm) was placed inside the milling jar. The milling tools were coated according to the standard procedure developed in our group.[1] The sealed reaction vessel was shaken for 10 minutes at 35 Hz in a MM500 mixer mill by *Retsch* at a temperature of 60°C. The crude product was dissolved in water and ethyl acetate and the water phase was extracted with ethyl acetate (2x20 mL). The organic phase was dried using $MgSO_4$ and the solvent was removed under vacuum. The product was quantified via HPLC. All reactions were conducted at least three times.

2.2 Finkelstein reaction

For the standard reaction, 4-Bromomethyl-1´,1´-Biphenyl (247 mg, 1 mmol, 1 eq.) and Sodium Iodide (449 mg, 3 mmol, 3 eq.) were placed in a 14 ml PFA vessel. Before the substrates were added, the milling ball (Ø 10 mm, 15 mm) was placed inside the milling jar. The reaction vessel was shaken for 5 minutes at 35 Hz in a MM500 mixer mill by *Retsch* at 60 °C. The crude product was dissolved in $CDCl_3$, the insoluble sodium salts were removed by filtering through a glass pipette filled with cotton and celite and the yield was determined by NMR by the ratio of starting material to product, as there are no side reactions occurring. All reactions were conducted at least three times.

3. Product characterization

4-Iodomethyl-1´,1´-Biphenyl

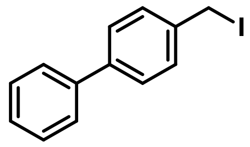

^{1}H NMR (500 MHz, $CDCl_3$) δ 7.51 (d, J = 8.6 Hz, 2H), 7.45 (d, J = 8.3 Hz, 2H), 7.39-7.35 (m, 4H), 7.28 (t, J = 7.3 Hz, 1H), 4.45 (s, 2H).

The yields presented in this study were determined by ^{1}H NMR spectroscopy. Figure S2 shows an example spectrum of the product mixture.

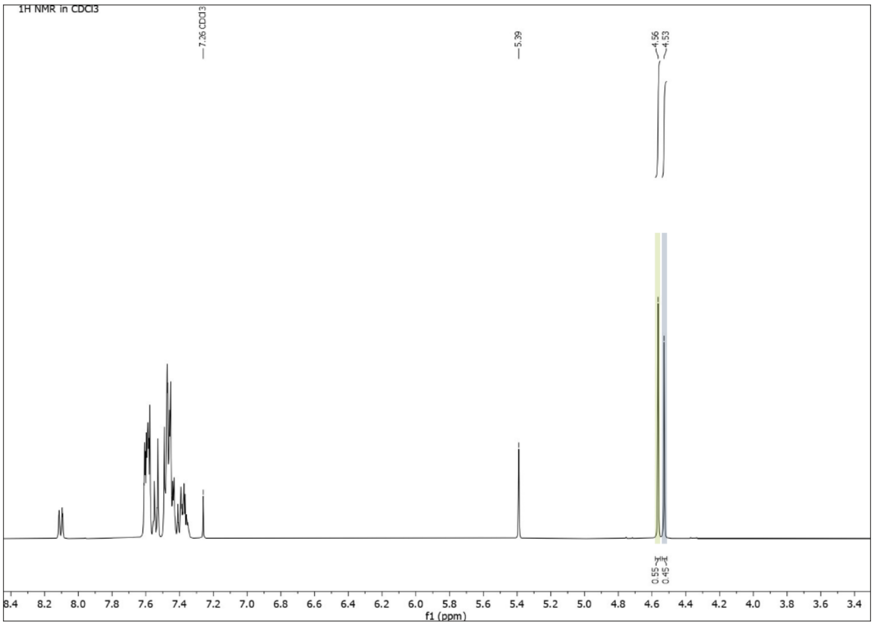

Figure S2: 1H NMR spectrum of a product mixture of a Finkelstein reaction conducted in this study. The signal of 4-Bromomethyl-1´,1´-Biphenyl (Substrate) is marked in green, and the Product signal is marked in blue. The signal at 5.3 ppm belongs to the standard Benzyl benzoate.

As no side reactions are expected under these conditions, the yields were calculated from the ratio of the product and starting material signals in the ^{1}H NMR spectra. Benzyl benzoate was used as an internal standard, since in some cases the solvent signal overlapped with the aromatic signals of both the starting material and the product.

4-Biphenylcarbaldehyde

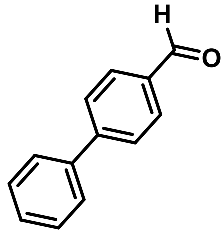

1H NMR (400 MHz, Methylene Chloride-d2) δ 10.05 (s, 1H), 8.03 – 7.91 (m, 2H), 7.82 – 7.75 (m, 2H), 7.72 – 7.63 (m, 2H), 7.49 (t, J = 7.5 Hz, 2H), 7.46 – 7.38 (m, 1H)

4. High-speed recordings

4.1 Equipment and Set-up

For the high-speed recordings a Sony RX100 VII with a frame rate of 1000 fps was used. All videos were recorded using a MM500 mixer mill by *Retsch,* and custom-made thin-walled milling vessels made from PFA. For the 40 ml vessel, a PMMA vessel was used to ensure sufficient observation of the movement. Furthermore, the vessel was illuminated using a lamp, which was mounted above the mill.

4.2 Observed movements

High-speed recordings were conducted of milling balls with a diameter of 10 mm, 15 mm and 20 mm. Two differently sized vessels were utilized, namely a 14 ml vessel and one with a volume of 30 mm. The latter was used to depict the movement of a 15 mm and a 20 mm milling ball, as it is approximately scaled for these milling ball diameters and can therefore be compared to the smaller scale approach of this work. Furthermore, the movement of regular cylinders, as well as cylinders with rounded ends was recorded at different milling frequencies. To observe the influence of the rheology of the reaction mixture on the ball movement, the reaction mixture of the Suzuki coupling and the Finkelstein reaction were added to the system and different time intervals were monitored. Figure 4 in the main manuscript shows the predominant movement paths observed for the different milling tools used.

5. Kinetic energy measurements and calculations

5.1 Kinetic energies derived from high-speed recordings

A schematic representation of the set up used for high-speed recordings is shown in Figure S3.

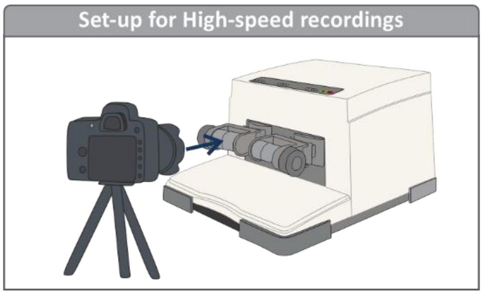

Figure S3: Schematic representation of the setup used for conducting the high-speed recordings. When referring to “the forward-facing wall”, this denotes the wall facing the camera.

The trajectories of the milling balls were analysed using the open-source software *Tracker Video Analysis and Modeling Tool*. High-speed recordings were imported at the original frame rate of the respective experiments (1000 frames per second (fps)). A known reference length – the outer length of the milling vessel – was used for spatial calibration within the software. The position of each milling ball was determined manually frame-by-frame over sequences of 500–1500 frames, depending on the visibility of the ball in the respective recording.

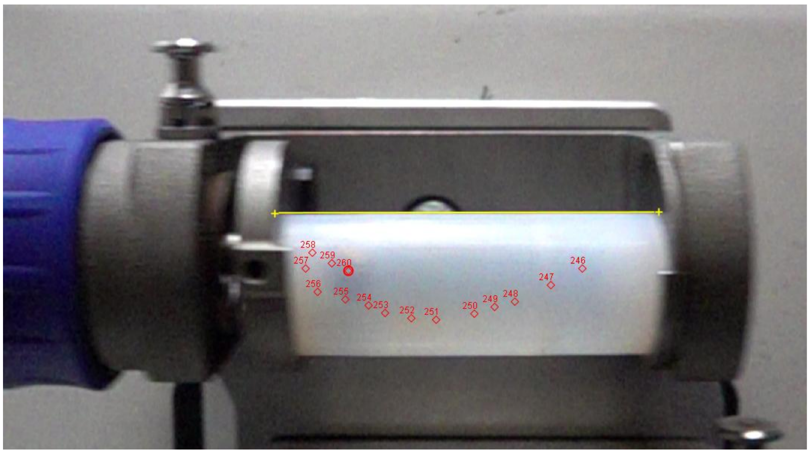

Figure S4: A frame from the *Tracker Video Analysis and Modeling Tool* illustrating the tracking of the ball position. The yellow line, corresponding to the known vessel length, was used as a reference to convert pixel distances into real distances. The ball center was manually marked in each frame, as the semi-translucent vessel limited visibility and prevented automatic tracking.

The calibrated x and y coordinates were exported and processed to calculate the displacement per frame. From these displacements and the known frame rate, the instantaneous velocities were obtained. For each trajectory, the mean velocity over the complete tracked sequence was calculated and used to determine the kinetic energy:

$$E_{kin} = \frac{1}{2} m \overline{v}^2 \quad (1)$$

where *m* is the mass of the respective milling ball and $\overline{v}$ the mean velocity. This approach reduces the influence of single-frame deviations or tracking inaccuracies and yields a representative kinetic energy value for the observed motion. In cases where the ball's motion included rolling components, this contribution was implicitly included in the measured displacement values.

It should be noted, however, that these measurements are not fully precise, as variations in the inclination of the holder may have slightly shortened the measured distances. Furthermore, certain points located at the ends of the vessel can be difficult to detect, as the PFA vessel used is only semi-transparent, which may have resulted in slight deviations as well. Additionally, the movement in the z-direction (along the viewing axis of the camera) cannot be observed, which may further account for slight differences of the kinetic energies shown.

The resulting kinetic energies of the milling balls at different frequencies are presented in Table S2:

Table S2: kinetic energies obtained by analysing the high-speed recordings conducted. Values were calculated for the differently sized hollow and solid milling balls inside the 14 ml, as well as 40 ml vessel.

Milling ball	Frequency/ Hz	E_{kin} (14 ml vessel) / mJ	E_{kin} (40 ml vessel) / mJ
10 mm hollow	35	12	
10 mm solid	35	40	
15 mm hollow	35	47	74
15 mm solid	35	68	149
20 mm hollow	35		122
20 mm solid	35		355
10 mm solid	30	32	
10 mm solid	25	12	
10 mm solid	20	7	

The results show that the decrease in kinetic energy for decreasing milling frequencies is not linear. This is depicted in Figure S5.

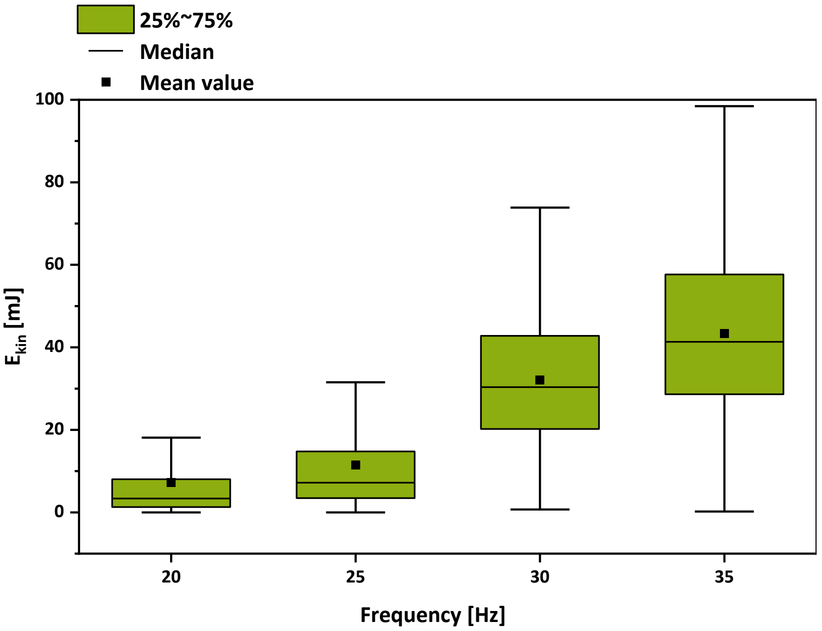

Figure S5: The mean kinetic energies derived from the high-speed recordings of the solid 10 mm bronze milling ball plotted against the milling frequencies applied. Frequencies of 20 Hz, 25 Hz, 30 Hz and 35 Hz were tested.

5.2 Calculated kinetic energies

Both trajectory models—the idealized linear (1D) motion and the figure-eight trajectory—represent simplified approximations of the actual ball dynamics in the mill. Both models should therefore be regarded as order-of-magnitude estimators that capture the principal dependencies of kinetic energy, for example on amplitude and frequency, while not fully describing the complex tribological and multi-body interactions present in real mechanochemical systems.

5.2.1 Idealized Figure of eight trajectory

To estimate the kinetic energy of a milling ball undergoing a figure-eight-like rolling motion inside a milling vessel in a MM500 mixer mill by *Retsch*, a time-resolved calculation was conducted over a duration of 2 seconds (corresponding to 70 oscillation cycles at 35 Hz). The calculation is based on the parametric form of the lemniscate of Bernoulli, which approximates the observed trajectory of the ball inside the vessel (Equation 1).

$$x = \frac{a \cdot cos\,(\theta)}{sin^2(\theta)+1} \text{ and } y = \frac{a \cdot sin(\theta)}{sin^2(\theta)+1} \qquad (2)$$

Here, θ defines the angular parameter over time *t*, with *f* = 35 Hz representing the milling frequency.

$$\theta = 2\pi f t \qquad (3)$$

The scaling parameter *a* was calculated from the maximum estimated displacements in the horizontal x_{max} and vertical y_{max} directions, using the equation:

$$a = \sqrt{\frac{(x_{max}^2 + y_{max}^2)^2}{2(x_{max}^2 - y_{max}^2)}} \qquad (4)$$

The values of x_{max} and y_{max} were based on the vessel's inner geometry. This choice reflects the maximum effective distance the milling ball can travel within the vessel during one oscillation cycle. While the milling vessel itself undergoes a sinusoidal motion, the ball predominantly moves along the vessel walls, and its motion remains confined within the physical limits of the vessel. Although the oscillation of the vessel slightly contributes to the total displacement of the ball in the laboratory reference frame, this contribution is minor compared to the overall travel range of the ball inside the vessel. Consequently, using half of the vessel length as the amplitude provides a realistic upper limit for the ball's effective trajectory.

The instantaneous velocity of the milling ball was obtained from the numerical gradient of its position:

$$v(t) = \sqrt{\left(\frac{dx}{dt}\right)^2 + \left(\frac{dy}{dt}\right)^2} \quad (5)$$

To be able to approximate the energy loss due to friction, an effective velocity v_{eff} can be calculated as follows:

$$v_{eff} = \begin{cases} \sqrt{v^2 - \mu k a_{max} \cdot |v \cdot \Delta t|} & if\ v^2 - \mu k a_{max} \geq 0 \\ 0 & otherwise \end{cases} \quad (6)$$

With the friction coefficient μ, which is set to μ=0 in this work, as losses due to friction are neglected. However, the equation is still shown including the friction coefficient to allow for its incorporation in future studies. The factor a_{max} corresponds to the estimated maximum vertical acceleration.

The kinetic energy at each time point was calculated using:

$$E_{kin}(t) = \frac{1}{2} m v_{eff}^2(t) \quad (7)$$

Where m=4.6g is the mass of the milling ball. The time-averaged kinetic energy $\langle E_{kin} \rangle$ was calculated according to:

$$\langle E_{kin} \rangle = \frac{1}{T} \int_0^T E_{kin}(t) dt \quad (8)$$

In practice, this integral was approximated numerically using the calculated values over N time steps:

$$\langle E_{kin} \rangle \approx \frac{1}{N} \sum_{i=1}^{N} E_{kin}(t_i) \quad (9)$$

5.2.2 Idealized straight movement

To estimate the kinetic energy of a milling ball undergoing idealized linear motion within an oscillating milling vessel, a numerical model was implemented in Python. The model assumes back-and-forth translation of the ball between the vessel walls and includes collision dynamics with energy dissipation.

Assumptions:

- The ball moves strictly in a linear path (1D), neglecting rotational motion and friction.
- Energy loss during collisions is neglected in this study, the coefficient of restitution was set to e=1. This was done, as the exact coefficient of restitution could not be determined in the course of this work. The equation is still shown including this coefficient to allow for its incorporation in future studies.
- A collision occurs after the vessel changes its direction of motion, as observed in the high-speed recordings using a 15 mm ball.
- The vessel's horizontal movement is modelled as sinusoidal with a frequency f=35 Hz and an amplitude A=1.25 cm.
- Time resolution is set to $\Delta t = 10^{-5}$ s, over a total time of 2 s.

The vessel is considered to oscillate sinusoidally:

$$x_{vessel}(t) = A \cdot sin(\omega t), \omega = 2\pi f \quad (10)$$

The vessel velocity is described as:

$$v_{vessel}(t) = \frac{dx_{vessel}(t)}{dt} = A \cdot \omega \cdot \cos(\omega t) \quad (11)$$

The effective free path available for the ball motion is given by the inner length of the jar minus the ball diameter:

$$L_{eff} = L_{vessel} - d_{ball} \quad (12)$$

Note: L_{eff} is a geometric limit (lab frame limits depend on jar position), while the *absolute* ball position in the laboratory frame is shown in equation 13. The ball's motion is computed numerically in small time steps Δt. Between collisions, the ball moves freely according to its current velocity:

$$x_{ball}(t+\Delta t) = x_{ball}(t) + v_{ball}(t)\cdot \Delta t \quad (13)$$

A collision with the vessel wall occurs when the relative distance between the ball and vessel exceeds half the available flight path:

$$|\Delta x(t)| = |x_{ball}(t) - x_{vessel}(t)| \geq \frac{L_{eff}}{2} \quad (14)$$

At the moment of collision, the velocity after impact is determined using the coefficient of restitution, which is set to e=1 in our study to reflect a collision without loss of energy:

$$v_{ball,new} = -e\cdot(v_{ball,old} - v_{vessel}) + v_{vessel} \quad (15)$$

Each time a collision occurs, the instantaneous kinetic energy is computed as:

$$\mathrm{E_{kin}} = \frac{1}{2}\mathrm{m}\cdot \mathrm{v_{ball}^2} \quad (16)$$

All such energy values are recorded over time, and at the end of the simulation, the mean kinetic energy is reported:

$$\langle E_{kin}\rangle \approx \frac{1}{N}\sum_{i=1}^{N} E_{kin,\mathrm{i}} \quad (17)$$

Where *N* is the number of collision events.

6. Suzuki coupling PA vessel vs. PFA vessel

For the Suzuki coupling of 4-iodobenzaldehyde and phenylboronic acid using hollow and solid milling balls of different sizes, a 14 mL polyamide vessel was used, as the previously employed PFA vessel could not be properly sealed within the heating jacket. Since the other reactions and the high-speed recordings were performed using a PFA vessel, the question arises whether comparable results are obtained for both vessel types. Figure S6 shows the results of a Suzuki coupling conducted in both vessel materials, demonstrating that the yields are similar.

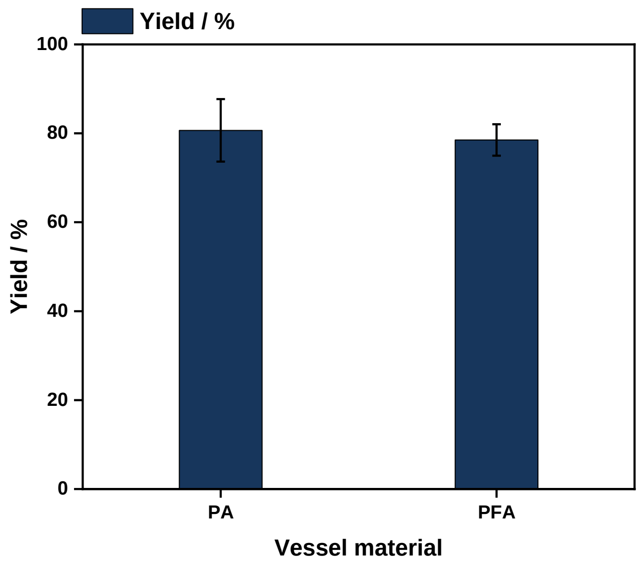

Figure S6: Yields of the Suzuki coupling with a PFA vessel compared to a PA vessel. The experiments were performed in a MM500 mixer mill by *Retsch* at a milling frequency of 35 Hz for 10 minutes at 60°C. A solid 10 mm milling ball and the reaction mixture (1 g K_2CO_3, 122 mg phenylboronic acid, 232 mg iodobenzene, 311 µl Butanol) were added to the respective vessel before milling.

Furthermore, it should be noted that the various screening experiments in this study — investigating, for instance, the effects of milling ball size were not quantitatively compared between different vessel types. Therefore, the use of different vessel materials does not affect the interpretation or validity of the conclusions drawn in this work.

7. Suzuki coupling – 40 mL vessel

High-speed recordings revealed that inside a 40 ml vessel, even balls with diameters of 15 mm and 20 mm follow the figure-eight shaped trajectory. Therefore, a comparison of hollow and solid milling balls of different sizes is of particular interest (Figure S7), as in this case contact time may play a more critical role than contact frequency.

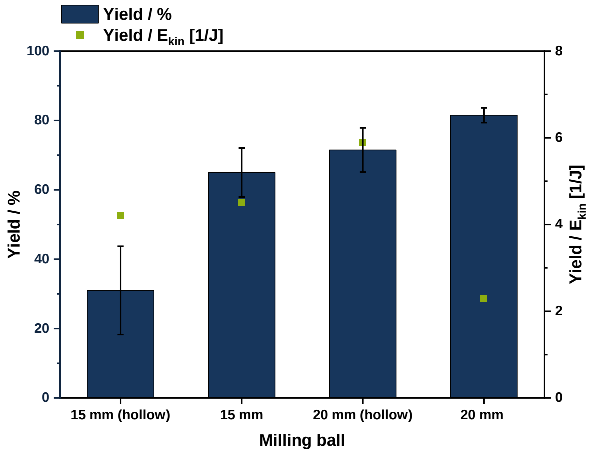

Figure S7: Yields, as well as the ratio of yield and kinetic energies for the larger vessel using the differently sized hollow and solid milling balls (Ø 15 mm and 20 mm). The experiments were performed in a MM500 mixer mill by Retsch at a milling frequency of 35 Hz for 5 minutes at 60°C. A 40 ml PFA vessel containing the milling material and the reaction mixture (2.8 g K_2CO_3, 348 mg phenylboronic acid, 662 mg iodobenzene, 888 µl Butanol) was utilized.

It is evident that the solid 20 mm ball performs inferior to the other balls tested. This may be attributed to the shorter contact time between the ball, the vessel wall, and the substrates, resulting from the higher velocity of the heavier ball. This effect may also explain why the hollow 20 mm ball performs slightly better than the solid 15 mm ball. The longer contact time could account for the comparable efficiency of the hollow and solid 15 mm balls as well, despite the hollow ball possessing only half the kinetic energy and exhibiting a similar movement pattern.

8. List of high-speed recordings

Vessel	Milling tool	Frequency / Hz	Substrate	File name
14 ml	10 mm ball	35 Hz	No	10mm_35Hz_14ml_vessel_without substrate
14 ml	10 mm ball	30 Hz	No	10mm_30Hz_14ml_vessel_without substrate
14 ml	10 mm ball	25 Hz	No	10mm_25Hz_14ml_vessel_without substrate
14 ml	10 mm ball	20 Hz	No	10mm_20Hz_14ml_vessel_without substrate
14 ml	10 mm ball, hollow	35 Hz	No	10mm_hollow_35Hz_14ml_vessel
14 ml	10 mm ball	35 Hz	Yes (with LAG)	10mm_35 Hz_14ml_vessel_with substrate_LAG_start
14 ml	10 mm ball	35 Hz	Yes (with LAG)	10mm_35 Hz_14ml_vessel_with substrate_LAG_20-30s
14 ml	10 mm	35 Hz	Yes (dry)	10mm_35Hz_14ml_vessel_with substrate_dry_start
14 ml	10 mm	35 Hz	Yes (dry)	10mm_35Hz_14ml_vessel_with substrate_dry_20-30s
14 ml	15 mm ball	35 Hz	No	15mm_35Hz_14ml_vessel
14 ml	15 mm ball	30 Hz	No	15mm_30Hz_14ml_vessel
14 ml	15 mm ball	25 Hz	No	15mm_25Hz_14ml_vessel
14 ml	15 mm ball	20 Hz	No	15mm_20Hz_14ml_vessel
14 ml	15 mm ball, hollow	35 Hz	No	15mm_hollow_35Hz_14ml_vessel
14 ml	Rounded cylinder (small)	35 Hz	No	Rounded cylinder_small_35Hz_14ml_vessel
14 ml	Rounded cylinder (large)	35 Hz	No	Rounded cylinder_large_35Hz_14ml_vessel
14 ml	Flat cylinder (small)	35 Hz	No	Flat cylinder_small_35Hz_14ml_vessel
14 ml	Flat cylinder (large)	35 Hz	No	Flat cylinder_large_35Hz_14ml_vessel
40 ml	15 mm ball	35 Hz	No	15mm_35Hz_40ml_vessel
40 ml	15 mm ball, hollow	35 Hz	No	15mm_hollow_35Hz_40ml_vessel
40 ml	20 mm ball	35 Hz	No	20mm_35Hz_40ml_vessel
40 ml	20 mm ball, hollow	35 Hz	No	20mm_hollow_35Hz_40ml_vessel
14 ml	10 mm ball	35 Hz	No	Modified vessel_flat inner ends_10mm_35Hz

References

1 M. Wohlgemuth, M. Mayer, M. Rappen, F. Schmidt, R. Saure, S. Grätz and L. Borchardt, From Inert to Catalytically Active Milling Media: Galvanostatic Coating for Direct Mechanocatalysis, *Angew. Chem., Int. Ed.*, 2022, **61**, e202212694.
